# Curing Behaviors, Mechanical and Dynamic Properties of Composites Containing Chloroprene and Butadiene Rubbers Crosslinked with Nano-Iron(III) Oxide

**DOI:** 10.3390/polym13060853

**Published:** 2021-03-10

**Authors:** Anna Słubik, Aleksandra Smejda-Krzewicka, Krzysztof Strzelec

**Affiliations:** Institute of Polymer and Dye Technology, Faculty of Chemistry, Lodz University of Technology, Stefanowskiego 12/16, 90-924 Lodz, Poland; aleksandra.smejda-krzewicka@p.lodz.pl (A.S.-K.); krzysztof.strzelec@p.lodz.pl (K.S.)

**Keywords:** elastomeric blends, nanocomposites, iron(III) oxide nanoparticles, flammability, Payne effect, Mullins effect

## Abstract

This paper discusses the curing behaviors, mechanical and dynamical properties of composites containing chloroprene rubber (CR) and butadiene rubber (BR) reinforced with mineral fillers. The iron(III) oxide nanoparticles were used as a crosslinking agent of the CR/BR blends. The research aimed to evaluate the effectiveness of nano-iron(III) oxide (nano-Fe_2_O_3_) as a new crosslinking agent while producing elastomeric materials with good mechanical properties and reduced flammability. The CR/BR (chloroprene rubber/butadiene rubber) blends were filled with silicas from natural resources (chalcedony, Neuburg silica earth) or silicas used in elastomer technology in many fields (aerosil, ultrasil). The results revealed that all composites were characterized by satisfactory tensile strength, tear resistance, and high resistance to fire. The filler dispersion in the elastomer matrix was carried out by using scanning electron microscopy (SEM), while the possibility of the filler–filler or filler–rubber interaction in the designed compositions was determined using the Payne effect and the Mullins effect.

## 1. Introduction

Polymer nanocomposites represent a class of materials that have assumed great importance in recent years and are the focus of extensive research. Nanocomposites are produced primarily as a result of introducing a nanometric substance into the polymer matrix. Small particle size and developed specific surface make it possible to obtain materials with specific properties, applicable in many fields. The main advantage of producing nanocomposites is obtaining materials with better properties (mechanical properties, thermal properties, flame retardation) compared to properties of the microcomposites [1,2].

Metal oxides with nanometric dimensions are an example of the substance involved in the production of elastomeric nanocomposites. Nano-sized iron(II,III) oxide was used as a filler for the ethylene-propylene rubber (EPM) or acrylonitrile-butadiene rubber (NBR). The incorporation of the nano-sized iron(II,III) oxide into the elastomeric matrix improves the mechanical properties of the obtained vulcanizates, additionally, this compound changes the magnetic properties [3]. The reinforcing properties of the nanomagnetic iron(II III) oxide were also observed in the case of the nanocomposites based on the recycled rubber modified by epoxy resin [4]. The use of iron(III) oxide nanoparticles as a filler of silicone rubber results in an improvement in the strength properties of products. Additionally, the nano-iron(III) oxide acts as dielectric permittivity. The improved properties of the composites make them suitable for applications in mechanical/electrical energy conversion [5]. The magnetorheological elastomers which can be effectively used as a base isolator in buildings or bridges to resist unpredictable loading conditions can be produced by filling silicone rubber with iron nanoparticles [6].

The nanocomposite based on the styrene-butadiene rubber (SBR) and copper nanoparticles were prepared on a laboratory rolling mill. Good dispersion of copper nanoparticles in the elastomeric matrix was determined by the transmission electron microscope and scanning electron microscope. Additionally, it was found that the incorporation of nanoparticles to the SBR slightly decreases the crosslinking density of the samples. The shear viscosity of the SBR matrix was increased with increasing the copper nanoparticles content. Adding the copper nanoparticles to the SBR results in improved mechanical properties as well [7]. The copper nanoparticles were also applied to obtain optimized mechanical and electrical properties of the nanocomposites based on the natural rubber cured with sulfur. It was found that the sample containing 0.074% of the copper nanoparticles possessed the highest electrical conductivity (0.014 S/m) at a percolation threshold and showed tensile strength around 13 MPa [8]. Whereas, the copper(II) oxide nanoparticles as nanofillers of the polyvinyl alcohol (PVA) have been investigated by Rao et al. The incorporation of the nanofiller into the PVA matrix improves the mechanical properties (elastic modulus, toughness, tensile strength). The sample containing 2.0 wt.% of copper(II) oxide nanoparticles was characterized by the best mechanical properties. The tensile strength for this composition increased by 50% while the toughness of the PVA composite increased by almost 137% compared to pure PVA [9].

The following nano-metal oxides, nano-zinc oxide (nano-ZnO), nano-aluminum oxide (nano-Al_2_O_3_), nano-zirconium oxide (nano-ZrO_2_) have been incorporated into the NBR and their effect on the rheological and structural properties of the vulcanizates was investigated. The activating ability of nano-sized metal oxide depends not only on particle size but also on the type of elastomeric matrix used, as well as on the type of crosslinking agent used. In this case, the di-sulfochloride benzene (DSCB) was used as a crosslinking agent. The DSCB activated nano-ZnO, which acts scavenger of hydrogen chloride released during the reaction of the elastomer and crosslinking agent. The zinc chloride is formed as a result of this reaction. The zinc chloride can activate crosslinking in the elastomer and participate in creating new bonds [10,11].

The effect of zinc oxide nanoparticles on crosslinking activity of the carboxylated acrylonitrile-butadiene rubber (XNBR) was investigated by Przybyszewska. In this work, the nano-zinc oxides with different specific surface areas, particle size and morphologies, as crosslinking agents of XNBR were used. For comparison purposes, the sample crosslinked by micrometric zinc oxide was also prepared. They found that the application of the zinc oxide nanoparticles led to producing the vulcanizates characterized by better mechanical properties and higher crosslinking density as compared to properties of the vulcanizates obtained by using the micro-sized zinc oxide. Additionally, the use of zinc oxide with nanometric dimensions allowed reducing the amount of micro-sized zinc oxide by 40% in the composition prepared by the standard way, while maintaining good properties of the vulcanizates obtained. The activity of the crosslinking process is mainly influenced by the morphology of the zinc oxide particles, while the specific surface and particle size have a minor impact on the effectiveness of the crosslinking agent [12]. The metal oxides nanoparticles are used in elastomer technology for the production of nanocomposites with satisfactory properties. The nano-metal oxides in nanocomposites usually act as fillers. There are also known nanocomposites, in which the metal oxide acts as a crosslinking agent.

The nanocomposites with satisfactory functional properties can be obtained after the incorporation of the nanofillers into the elastomer matrix. The addition of the nano-silica to the blend of ethylene-propylene-diene rubber (EPDM) and chloroprene rubber (CR) not only improves the mechanical properties of the obtained vulcanizates but also increases the compatibility between the rubbers used. Based on the SEM images, the presence of nano-silica in the EPDM and the CR phases was observed. Increased compatibility between the ingredients in the blends results in improved modulus, tensile strength, and thermal properties of the samples [13].

The article by Lim et al. describes the preparation of the CR/nano-silica composites. It was found that the hydrophobicity and the hardness of the CR/nano-silica composite increased as the silica content was increased. Additionally, the sample containing 4 phr of nano-silica was characterized by the most uniform dispersion state and the highest peel strength [14].

Silicon rubber composites filled with nano-silica are now widely used as high voltage insulating materials in power transmission and substation systems. In the work of Wu et al. the influence of pyrolytic nano-silica on the dielectric and mechanical properties of silicone rubber was investigated. In the case of the surface modified nano-silica composite, a good dispersion of the filler particles was observed. Good dispersion of the filler in the elastomer matrix led to a uniform stress distribution during stretching, resulting in greater elongation at break. The tensile strength (TS_b_) of vulcanizates did not depend on the type of filler, as evidenced by the similar TS_b_ value of both types of composites [15].

Our research aimed to design elastomeric composites containing chloroprene rubber and butadiene rubber crosslinked with nano-iron(III) oxide. Our previous research [16] confirmed the possibility of crosslinking the CR/BR blends with micrometric iron(III) oxide. Therefore, in this article, we decided to use the nano-iron(III) oxide as a crosslinking agent for the CR/BR blends and to investigate the effect of the nano-Fe_2_O_3_ on the functional properties of the obtained products. Additionally, the CR/BR blends were filled with silicas from natural resources (chalcedony, Neuburg silica earth) or silicas (aerosil, ultrasil) used in elastomer technology in many fields.

## 2. Materials and Methods

### 2.1. Materials

The object of the research was an elastomeric blend containing chloroprene rubber and butadiene rubber. The detailed characteristics of rubbers are presented in Table 1.

As a crosslinking agent, the nano-iron(III) oxide (nano-Fe_2_O_3_) from Sigma Aldrich Chemical Co. (Darmstadt, Germany) was used. Stearic acid (Chemical Worldwide Business Sp. z o. o. Słupca, Poland) was used as a dispersing agent.

The elastomeric blends were filled with the following fillers (Table 2):

### 2.2. Preparation and Vulcanization of the Rubber Blends

The rubber blends with the composition given in Table 3 were prepared with a laboratory two-roll-mill with rolls of the following dimensions: diameter—150 mm and length—300 mm. The constituents were added in the sequence presented in Table 3. First, the rubbers were plasticized, then the stearic acid and the filler (aerosil, ultrasil, chalcedony, or sillitin) were incorporated. Finally, the crosslinking agent (nano-Fe_2_O_3_) was added Preparation of the unfilled blend took 6 min, while the filled blends took about 12 min.

Then the prepared samples were vulcanized using an electrically heated hydraulic press. The vulcanization parameters were as follows: temperature—160 °C, pressure—150 bars, time—15 min for unfilled sample and sample filled with ultrasil, chalcedony or sillitin, 30 min for sample filled with aerosil.

### 2.3. Dynamic Properties

To investigate the dynamic properties of vulcanizates, the Payne effect was determined based on the storage modulus (G′) and the loss modulus (G″). The determination was made using the Ares G2 rheometer (New Castle, DE, USA). Disc-shaped vulcanizates with a thickness of ~2 mm were used for the measurement. The sample was then placed between the measuring plates of the apparatus, and the clamping force was 10 N. The measurement was carried out at room temperature.

### 2.4. Hysteresis Losses and Mullins Effect

The hysteresis losses were carried out by using a Zwick testing machine, model 1435 (Ulm, Germany). Three paddle-samples were used for this measurement. The samples were stretched five times to an elongation of 200%. The stretching speed was 500 mm/min, and the initial force was 0.1 N. Based on the results obtained, the Mullins effect was determined according to the following formula (Equation (1)):

(1) $$E_{M}=\frac{W_{1}-W_{5}}{W_{1}}\times 100\%$$

where: W_1_ is hysteresis loss at the first extension of the sample (kJ/m^2^), W_5_ is hysteresis loss at the fifth extension of the sample (kJ/m^2^).

### 2.5. Differential Scanning Calorimetry

The thermal changes of unfilled and filled CR/BR/nano-Fe_2_O_3_ vulcanizates were carried out by differential scanning calorimetry (DSC), using a model DSC1 Mettler Toledo (Greifensee, Switzeland). The operating temperature of the DSC analysis was within a range of −150–250 °C at a constant heating rate of 10 °C/min, whereas the liquid nitrogen was used as the coolant.

### 2.6. Morphology Studies

The surface morphology and cross-section of the polymeric samples were assessed using a scanning electron microscope (SEM) by Hitachi Tabletop Microscope TM-1000 (Tokyo, Japan). Preparation of samples for measurement consisted in placing a double-sided self-adhesive foil on special tables and gluing the tested sample to it. The cross-section of the sample was obtained by cutting the sample with a scalpel. Then, a gold layer using the Cressington Sputter coater 108 auto vacuum sputtering machine (Redding, CA, USA) at a pressure over 40 mbar, for 60 s was applied to the sample prepared. The sample prepared in this way was placed in a scanning electron microscope chamber and the measurement was performed. Using the TM-1000 software, the results were recorded on a computer cooperating with the spectrophotometer.

### 2.7. Cure Characteristic

The crosslinking kinetics of the tested elastomeric blends were determined with an Alpha MDR 2000 rheometer (Hudson, NY, USA) according to ASTM D5289-17 standard. The test consisted of registering the torque M as a function of time t during the crosslinking of the test sample at a constant temperature T. The torque value depends on the stiffness of the blend and changes as the crosslinking process progresses. Based on the vulcametric curves M = f (t), the optimal cross-linking time t_90_ (the time in which the torque reaches 90% of the increase), the scorch time (t_o2_), the minimum torque (M_min_) and increment of torque after a specified time of heating (ΔMt) were determined. The cure rate index (CRI), a measure of the rate of curing, was calculated using the Equation (2):

(2) $$\mathrm{CRI}=\;\frac{100}{t_{90}-t_{02}}$$

The equilibrium swelling was determined in the presence of toluene or heptane. From the vulcanizates, four samples of 30–50 mg of different shapes were cut out, which, after weighing on an analytical balance with an accuracy of 0.1 mg, were placed in weighing vessels and poured with the solvent. The swelling process took place at room temperature for 72 h. This is the time sufficient for the process to reach the equilibrium state. After this time, the swollen samples were removed from the solvent, their surface was dried with filter paper and weighed on an analytical balance. Then the samples were dried to a constant weight at T = 50 °C and they were reweighed. The equilibrium swelling degree in toluene or heptane (Q_v_) was calculated based on the equation (Equation (3)):

(3) $$Q_{v}={\;Q}_{w}\times \frac{d_{v}}{d_{s}}$$

where: Q_w_ is the value of equilibrium mass swelling (mg/mg); d_v_ is vulcanizate density (g/cm^2^), d_s_ is solvent density (g/cm^2^).

### 2.8. Mechanical Test

The tensile strength properties were measured in accordance with the PN-ISO 37: 2007 standard using a ZWICK machine (model 1435) (Ulm, Germany) connected with the appropriate computer software. In the study, samples in the shape of B-type paddles with a measuring section width of 4 mm were used. The scope of the properties tests was included: stress at 100%, 200%, 300% of elongation (S_e100_, S_e200_, S_e300_), tensile strength (TS_b_), and elongation at break (E_b_).

The tear strength (T_s_) was measured using the A method in accordance with the PN-ISO 34-1: 2010 standard using a ZWICK machine (model 1435) connected with the appropriate computer software. Rectangular-shaped samples with the following dimensions 100 × 15 mm^2^ were used for the tests.

The hardness (HA) was measured with a Zwick/Roell (Ulm, Germany) hardness tester in accordance with ISO-48-4: 2018. The samples for this test were prepared in the shape of cylinders in a specially prepared form. The measurement result was determined on the Shore A scale.

The measurement of damping properties under specified stress conditions was performed with the ZWICK ZMARTPRO 1435 testing machine by ZWICK/Roell (Ulm, Germany). In this study, the cylindrical samples with a diameter of 35.0 mm and a height of 17.8 mm, were used. The value of the maximum cyclic stress depended on the hardness of the vulcanizate (0.5 MPa for samples with a hardness of 30–40 °Sh A; 0.7 MPa for samples with a hardness of 41–50 °Sh A; 1.2 MPa for samples with a hardness of 51–65 °Sh A; 2.0 MPa for samples with a hardness of 66–85 °Sh A). Based on the obtained results, the value of the relative damping (T_τw_) was calculated according to the equation (Equation (4)):

(4) $$T_{\tau w}=\frac{{\Delta W}_{i}}{W_{\mathrm{ibel}}}\times 100\%$$

where: ΔW_i_ is the difference between the work of compression and the work while reducing the strain (Nmm), W_ibel_ is work of compression (Nmm).

### 2.9. Thermal Aging

The samples for the measurement of thermooxidative aging were placed in the drying chamber, heated to the temperature of 70 °C for 7 days. After another day, the strength properties (stress at elongation of 100%, 200%, 300%, tensile strength, elongation at break) of the samples were studied. Based on the aging index (K), changes of mechanical properties caused by thermooxidative aging were evaluated. Aging index was determined in Equation (5):

(5) $$K=\;\frac{{\mathrm{TS}}_{b}^{\prime }\times E_{b}^{\prime }}{{\mathrm{TS}}_{b}\cdot E_{b}}$$

where: TS_b_ is the tensile strength before thermooxidative aging (MP), TS_b_’ is the tensile strength after thermooxidative aging (MPa), E_b_ is the elongation at break before thermooxidative aging (%), E_b_’ is the elongation at break after thermooxidative aging (%).

### 2.10. Flammability

The flammability of the obtained vulcanizates was determined using the oxygen index method. For the test, samples with dimensions of 50 × 10 × 4 mm^3^ were prepared. The samples were mounted in a holder that was covered with a quartz column. Then the gas flow rate in which the sample was washed was determined. The nitrogen flow rate was 400 L/h, and the oxygen flow rate was adjusted to determine its lowest concentration, in the mixture of oxygen and nitrogen, at which the sample burned completely in 180 ± 10 s. The oxygen index (OI) value was calculated based on the following equation (Equation (6)):

(6) $$\mathrm{OI}=\;\frac{O_{2}}{O_{2}+N_{2}}\times 100\%$$

where: O_2_ is oxygen flow rate (L/h), N_2_ is nitrogen flow rate (L/h).

### 2.11. Thermogravimetry Analysis and First Derivative Thermogravimetry

The thermogravimetry analysis (TGA) and first derivative thermogravimetry (DTG) analyses were performed using a two-step procedure, using a model DSC1 Mettler Toledo (Greifensee, Switzeland). First, samples of composition were heated in the temperature range of 25–600 °C in an argon atmosphere with a heating rate of 20 °C/min. Next, the gas was changed into the air and heating was continued up to 900 °C with the same heating rate.

## 3. Results and Discussion

### 3.1. Morphology Analysis of Unfilled and Filled CR/BR/Nano-Fe_2_O_3_ Vulcanizates

**Figure 1:**
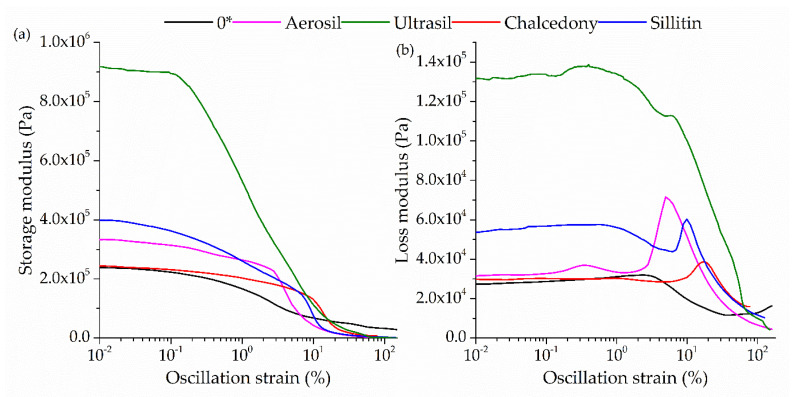
Storage modulus (**a**) and loss modulus (**b**) of unfilled and filled CR/BR/nano-Fe_2_O_3_ vulcanizates.

The results from the measurement of the dynamic properties are presented in Table 4 and in Figure 1. Dynamic tests of vulcanizates carried out under conditions of constant temperature, constant deformation frequency, and changing deformation amplitude show that the storage modulus decreases with increasing deformation amplitude. This phenomenon is known as the Payne effect. The highest Payne effect was obtained for vulcanizate filled with ultrasil (ΔG = 898,076 Pa), which proves the possibility of creating a structure by the filler particles. Additionally, the ultrasil particle can form agglomerates or aggregates, as indicated also by the maximum value of the storage modulus. The more developed the filler network, the higher the value of the storage modulus. In the case of the vulcanizate filled with aerosil, the Payne effect was 332,015 Pa. The low value of the Payne effects in the case of a sample filled with aerosil, which belongs to active fillers, may indicate good filler dispersion in the elastomeric matrix, as well as the possibility of filler–filler or filler–rubber interactions. Whereas, the incorporation of the silica from natural sources (chalcedony or sillitin) into the CR/BR blend increased the storage modulus.

The value of the loss modulus, and thus the amount of energy dissipated during the dynamic deformation of the sample, depends on two processes: destroying and rebuilding the structure of the filler network. The greater part of the filler network is destroyed and rebuilt during one deformation cycle, the greater the value of the loss modulus. The highest value of the loss modulus was obtained for a sample filled with ultrasil, which confirms the possibility of the formation of the filler network in the elastomeric matrix. The formation of the structure by the filler in the elastomeric matrix or the presence of filler–rubber interactions affect the morphology, degree of crosslinking, and mechanical properties of the prepared compositions.

The Mullins effect provides information about the filler–filler and filler–rubber interaction. Based on the results obtained from the hysteresis test under static tensile conditions (Table 5) it was found that the formation of additional bonds between CR macromolecules and silica or filler–filler interactions may take place in the sample filled with aerosil. The ΔW_i_ value for this vulcanizate was 550.8 Nmm. Such a high value of ΔW_i_ may suggest the presence of rubber–filler interactions, in which the destruction requires more energy than the destruction of the filler network. The ΔW_i_ value for the sample with the highest Payne effect (CR/BR/ultrasil) was 179.0 Nmm, which confirms the interactions between the particle of this filler. For comparison, the hysteresis losses for the unfilled vulcanizate were only 38.1 Nmm, while the ΔW_i_ value for the vulcanizate containing chalcedony was 86.3 Nmm and for the sample filled with sillitin was 90.3 Nmm. The Mullins effect for the composition filled with chalcedony or sillitin was 27.5% and 45.3%, respectively. The greater Mullins effect in the vulcanizate containing aerosil or ultrasil was due to the more developed active surface of these fillers and consequently, the stronger filler–rubber or filler–filler interactions.

**Figure 2:**
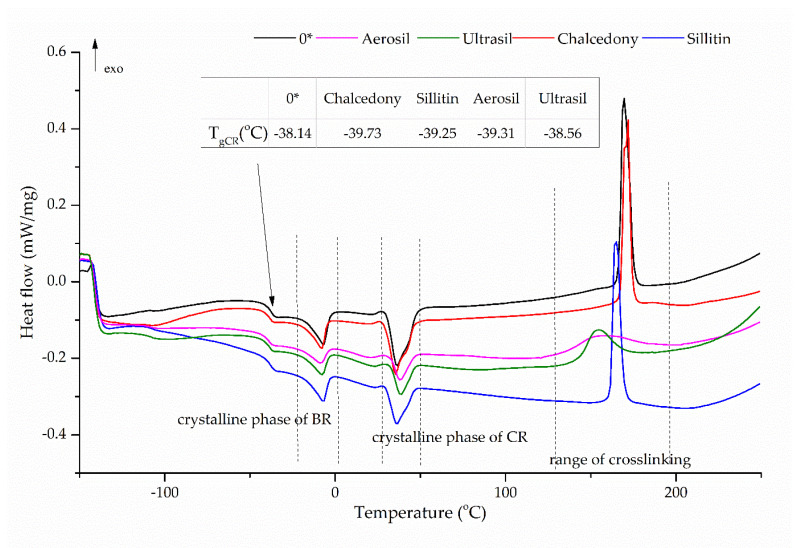
DSC of unfilled and filled CR/BR/nano-Fe_2_O_3_ blends.

The thermal properties of unfilled and filled (aerosil, ultrasil, chalcedony, and sillitin) CR/BR composites are shown in Figure 2. The incorporation of the filler into the CR/BR matrix causes changes in the peaks coming from the CR and BR crystallization phases, as well as within the range corresponding to crosslinking range. In the case of the sample filled with aerosil and ultrasil, a clear reduction of the CR and BR crystalline phase was observed. This reduction of the crystalline phases may be due to the presence of hydrogen bonds between the filler particles and the consequent formation of the internal structure by the filler, which hiders the orientation of the elastomer chains, reduces the order of the system, hindering crystallization (confirmed by results from the Payne effect and the Mullins effect). In the case of samples filled with aerosil or ultrasil, the shift of the melting peak of the CR crystalline phase towards higher temperature was observed. The smaller changes in the CR and BR crystalline phase after the incorporation of chalcedony or sillitin into the CR/BR blends proved the lower ability of these fillers to create the filler network. Additionally, the presence of sillitin, aerosil, or ultrasil in the blends caused the exothermic peak corresponding to the crosslinking to shift towards a lower temperature.

**Figure 3:**
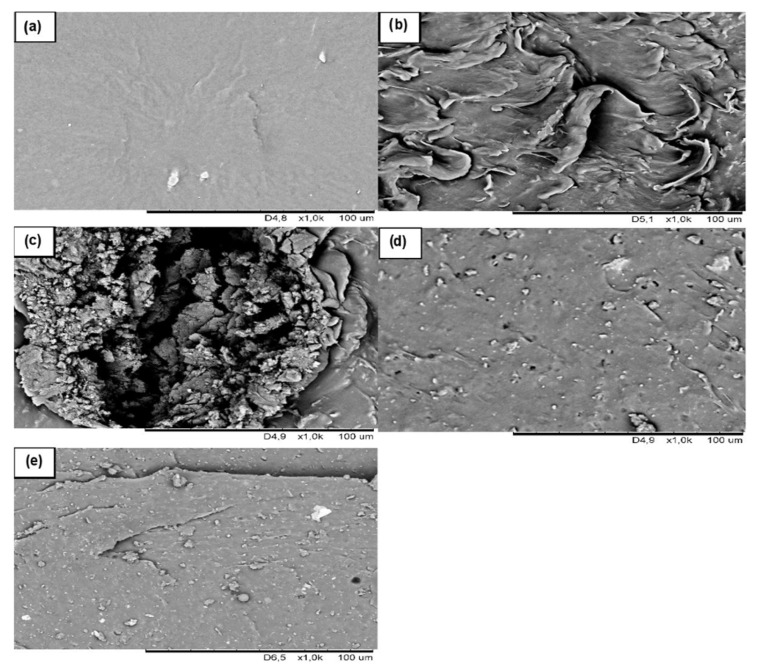
SEM images of the cross-section of: CR/BR/nano-Fe_2_O_3_ (**a**); CR/BR/aerosil (**b**); CR/BR/ultrasil (**c**); CR/BR/chalcedony (**d**); CR/BR/sillitin (**e**).

The cross-section surface morphology of the obtained vulcanizates is shown in Figure 3. The surface of the unfilled sample (Figure 3a) was homogeneous, which proves good compatibility of both rubbers in the blend crosslinked with nano-iron(III) oxide. The incorporation of the filler changes the surface of the resulting compositions. The presence of aerosil in the CR/BR vulcanizate resulted in a rough surface with numerous grooves (Figure 3b). Although this filler has the most developed specific surface area (BET_aerosil_ = 380 m^2^/g) and the smallest particle size, it was well dispersed in the elastomeric matrix since agglomerates or aggregates were not observed. Figure 3c shows SEM images of samples filled with ultrasil. In the center of the picture, a round particle larger than 100 nm with voids was observed. This particle was probably an agglomerate of the filler. Ultrasil can form aggregates and agglomerates, which was confirmed on the basis of dynamic results (Figure 1, Table 4). The chalcedony and the sillitin have a similar specific surface area (BET_chalcedony_ = 10 m^2^/g, BET_sillitin_ = 12 m^2^/g) and a similar particle size equal to 9 and 13 µm, respectively. Figure 3d,e shows the filler particles with a size less than 10 µm evenly distributed throughout the elastomer matrix.

**Figure 4:**
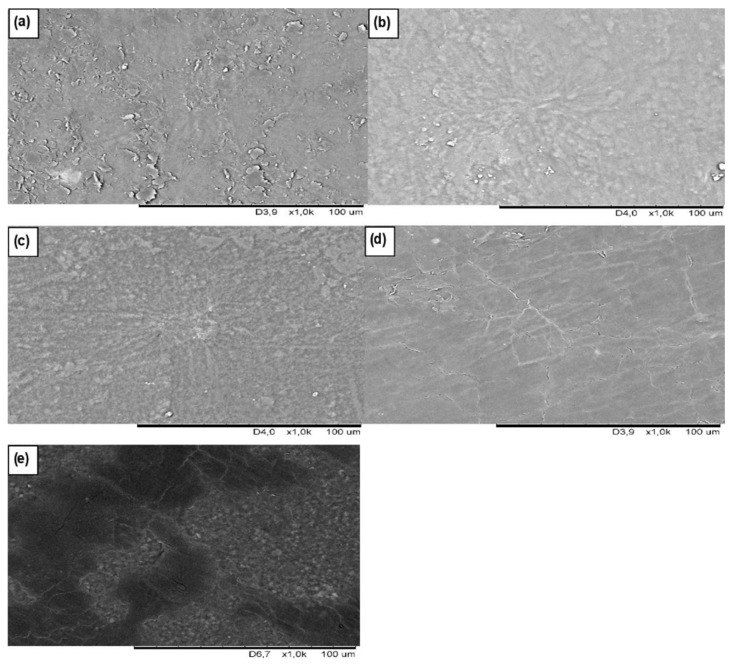
SEM images of the surface of: CR/BR/nano-Fe_2_O_3_ (**a**); CR/BR/aerosil (**b**); CR/BR/ultrasil (**c**); CR/BR/chalcedony (**d**); CR/BR/sillitin (**e**).

The surface morphology of the tested vulcanizate is shown in Figure 4. The surface of the unfilled sample was rough (Figure 4a). The sample containing aerosil had morphology similar to the sample filled with ultrasil (Figure 4b,c). Whereas, the composition filled with chalcedony had numerous cracks on its surface (Figure 4d). In the case of the vulcanizate containing sillitin, a heterogeneous surface with darker (elastomeric matrix) and lighter areas (probably filler agglomerates) was observed (Figure 4e).

### 3.2. Cure Characteristic of Unfilled and Filled CR/BR/Nano-Fe_2_O_3_ Composites

**Figure 5:**
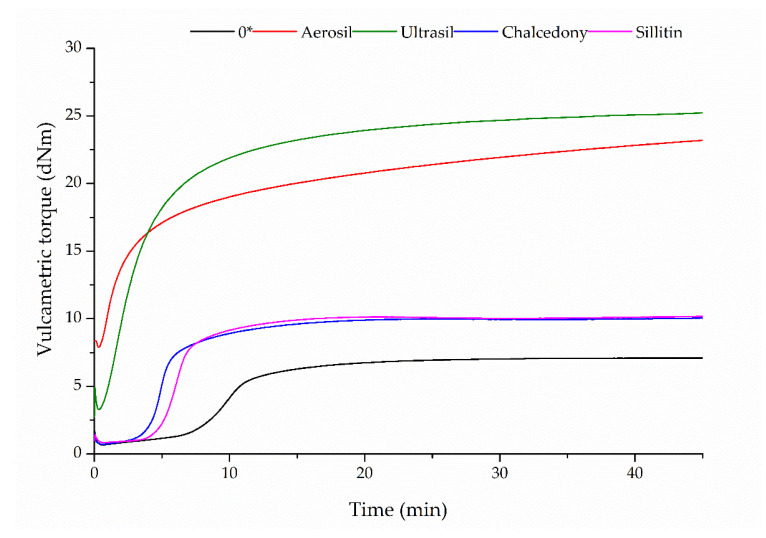
The rheometric curves of unfilled and filled CR/BR/nano-Fe_2_O_3_ blends.

The use of nano-iron(III) oxide enables the crosslinking of the blend containing chloroprene rubber and butadiene rubber. The CR/BR/nano-Fe_2_O_3_ blend was characterized by the following vulcametric parameters: t_02_ = 7.30 min, t_90_ = 16.70 min, M_min_ = 0.68 dNm, ΔM_30_ = 5.93 dNm (Table 6, Figure 5) Comparing the properties of the CR/BR blends crosslinked with nano-Fe_2_O_3_ to the properties of CR/BR/micro-Fe_2_O_3_ composition [16], the positive effect of nano-Fe_2_O_3_ was observed in the case of the scorch time. For the CR/BR/nano-Fe_2_O_3_ sample, the value of this parameter was 7.30 min. Whereas, the use of micro-sized iron(III) oxide led to a reduction in the scorch time (t_02_ = 3.20 min). The inverse dependency was observed for the vulcanization time. The t_90_ value for the CR/BR blend crosslinked with nano-Fe_2_O_3_ was longer than for the CR/BR composition crosslinked with micro-Fe_2_O_3_. The use of nano-iron(III) oxide as a crosslinking agent for the CR/BR blends had a comparable effect on the crosslinking degree, as the use of the micro-sized iron(III) oxides. The equilibrium swelling value in toluene for CR/BR/nano-Fe_2_O_3_ was 6.20 mL/mL, while Q_v_^T^ value for the CR/BR/micro-Fe_2_O_3_ was 5.97 mL/mL [16].

**Scheme 1:**
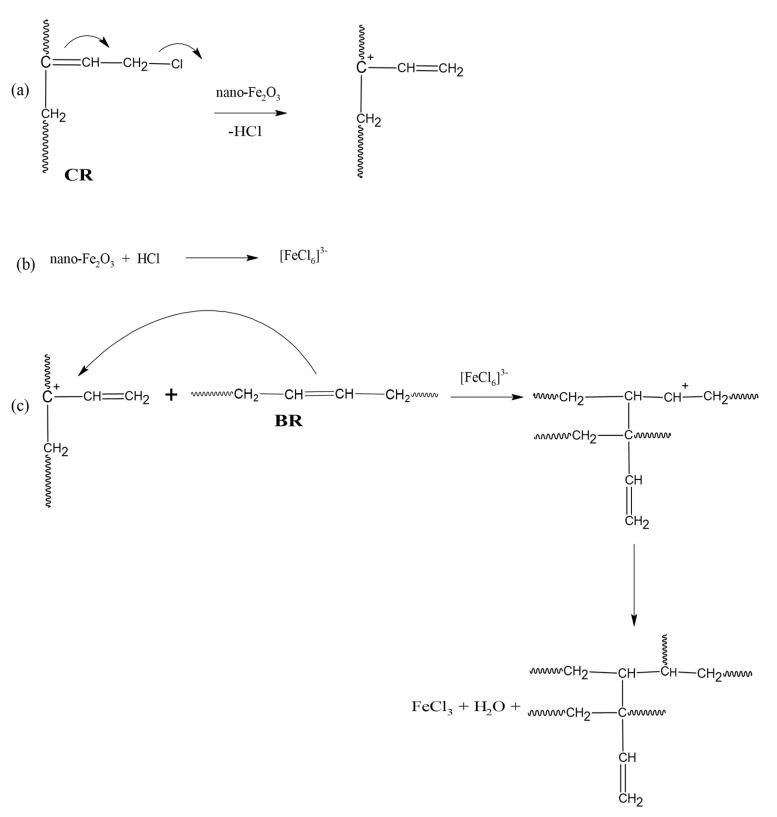
Crosslinking mechanism of CR/BR blends in the presence of iron(III) oxide nanoparticles: (**a**) dehydrohalogenation of chloroprene rubber, (**b**) formation of the iron-chloride complex and (**c**) formation of interelastomeric bond between chloroprene rubber and butadiene rubber.

The use of nano-iron(III) oxide as a crosslinking agent of CR/BR blends, probably leads to the formation of an interelastomeric bond between the tested rubbers. The proposed crosslinking mechanism of the CR/BR composites in the presence of iron(III) oxide nanoparticles is shown in Scheme 1.

Crosslinking of the CR/BR blends in the presence of iron(III) oxide nanoparticles (Scheme 1) begins with dehydrohalogenation of chloroprene rubber with the release of hydrogen chloride (HCl). Subsequently, iron(III) oxide nanoparticles react with the hydrogen chloride to form the iron-chloride complex ([FeCl_6_]^3−^), which acts as a catalyst necessary for the formation of the interelastomeric bond between chloroprene rubber and butadiene rubber. The detachment of the chlorine atom from CR macromolecules leads to the formation of the carbocation. Then the carbocation reacts with the double bond derived from BR, which leads to the formation of the interelastomeric bond between rubbers. The use of the following metal oxides (Cu_2_O, CuO, or Fe_2_O_3_) as a crosslinking agent also leads to the interelastomeric connection of chloroprene rubber with butadiene rubber. A detailed description of the CR/BR crosslinking mechanism using the other metal oxides has been described in our previous articles [16,17,18].

In the case of filled blends, the incorporation of the chalcedony to the chloroprene and butadiene rubber blends had a comparable effect on the vulcametric parameters as the addition of the sillitin into the tested composition as evidence by the similar values of the scorch time, the cure time, minimum torque, and increment of torque after heating (Figure 5, Table 6). Significant differences in the described parameters were observed after using aerosil as a filler incorporated into the CR/BR composition.

The viscosity of the elastomer blends was determined by the minimal torque. For the sample containing aerosil the M_min_ value was 6.17 dNm, whereas the samples with silicas derived from natural sources were characterized by a lower value of this parameter (0.72 or 0.84 dNm for chalcedony and sillitin, respectively). The highest viscosity of the CR/BR blend filled with aerosil results from the high specific surface area of the filler used (BET = 380 m^2^/g). The intermediate value of this parameter was obtained for composition filled with ultrasil.

The scorch time for the sample containing aerosil was only 0.06 min, which may indicate the presence of hydrogen interactions between the silanol group on the silica surface and the chlorine atom derived from the CR macromolecule. Such interaction may occur already at the stage of preparing the blend, leading to the reduction of the scorch time [19]. In the case of the remaining compositions, the scorch time was 1.10 min (ultrasil), 3.78 min (chalcedony), and 4.70 min (sillitin).

The CR/BR blend filled with ultrasil was characterized by the higher increase of torque after 30 min of heating (ΔM_30_ = 21.49 dNm). The CR/BR/aerosil blend had an almost two times higher value of the ΔM_30_ as compared to the sample filled with chalcedony or sillitin. Aerosil and ultrasil belong to the active fillers, whereas the chalcedony and sillitin are classified as passive fillers. The more developed specific surface area, as well as the possibility of chemical bonds at elevated temperature reactions between the silanol groups present on the silica surface and the chlorine atom located in the chloroprene rubber leading to creating a new covalent bond, may contribute to an increase in the degree of crosslinking of the elastomer composition prepared.

According to the result obtained from the measurement of the equilibrium swelling (Table 6) it was found that the composition filled with aerosil showed increased resistance to the organic solvent (toluene or heptane). The vulcanizate containing ultrasil had the same equilibrium swelling value as the reference sample. Whereas, the incorporation of chalcedony or sillitin to the CR/BR elastomer matrix led to an increase in the Q_V_^T^ to the value of ~6.64 mL/mL.

### 3.3. Mechanical Properties of Unfilled and Filled CR/BR/Nano-Fe_2_O_3_ Vulcanizates

The strength properties of unfilled and filled CR/BR/nano-Fe_2_O_3_ vulcanizates are presented in Table 7. The stress at 100%, 200%, and 300% elongation for the unfilled CR/BR/nano-Fe_2_O_3_ composition was 0.70, 0.99, and 1.28 MPa, respectively. The use of micro-sized iron(III) oxide had a comparable effect on these parameters Se_100_ = 0.65 MPa, Se_200_ = 0.96 MPa, Se_300_ = 1.26 MPa) [16]. Other strength parameters also do not significantly depend on the type of iron(III) oxide used. The tensile strength (TS_b_ = 10.20 MPa) of the CR/BR/micro-Fe_2_O_3_ was slightly greater than the tensile strength (TS_b_ = 9.76 MPa) of the CR/BR/nano-Fe_2_O_3_ sample. The CR/BR composition crosslinked with nano-Fe_2_O_3_ was characterized by elongation at a break equal to 1120%, while for the CR/BR/micro-Fe_2_O_3_ sample, the E_b_ value was 816% [16].

Rigid aerosil particles contribute to the production of rigid vulcanizates, as evidenced by the highest stress value at 100%, 200%, and 300% of elongation, the lowest value of the relative elongation at break (650%), and the highest hardness (HA = 65.1 °ShA). In the case of tensile strength, the sample containing aerosil had the highest value of this parameter (TS_b_ = 19.80 MPa). It is interesting that the vulcanizate filled with chalcedony or sillitin, having a comparable degree of crosslinking determined based on vulcametric tests and equilibrium swelling, differed significantly in their strength properties. The tensile strength for CR/BR/sillitin vulcanizate was almost two times higher than the sample containing chalcedony, which may be due to the different shape and size of the filler particle. The chalcedony is a mineral with a large particle size distribution, which may result in local loads on the elastomeric matrix caused by large filler particles. Such defects can act as microcracks, weakening the material and deteriorating its mechanical properties, which was observed in the SEM photos (Figure 4d) [20]. The composition filled with ultrasil was characterized by the worst strength properties, which may be caused by the filler creating agglomerates and aggregates that constitute local loads (SEM image—Figure 3c).

The tear resistance increases with the incorporation of the filler (Table 8). The greatest force needed to tear the material should be applied to the sample containing aerosil (T_s_ = 12.60 N/mm), while the lowest for the vulcanizate filled with chalcedony (T_s_ = 3.93 N/mm).

In the case of the relative damping for the unfilled CR/BR vulcanizate, it reached a value equal to 10.00% (Table 8). The addition of filler into the CR/BR matrix led to an improvement in the damping properties (T_τw_ was from 25.83% to 31.05%). The higher the relative damping value, the more the material is able to minimize vibration.

**Figure 6:**
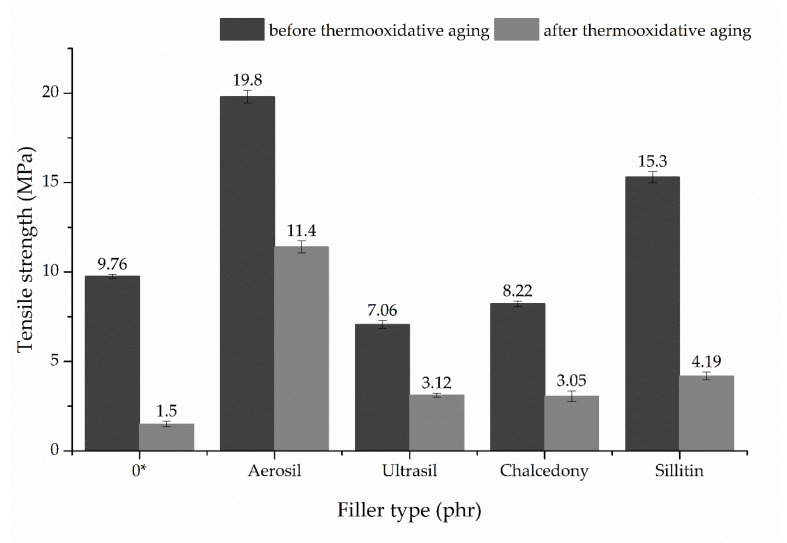
Mechanical properties of unfilled and filled CR/BR/nano-Fe_2_O_3_ vulcanizates, before and after thermooxidative aging.

Analyzing the results of the aging tests, it was found that the vulcanizates showed poor resistance to aging factors, regardless of the composition of the tested products (Figure 6). The tensile strength was reduced for the CR/BR/aerosil sample from 19.80 to 11.40 MPa, for CR/BR/ultrasil from 7.06 to 3.12 MPa, for the CR/BR/chalcedony vulcanizate from 8.22 to 3.05 MPa, and for the CR/BR/sillitin sample from 15.30 to 4.19 MPa. The elongation at break also deteriorated significantly as a result of the aging factors. For the unfilled sample, the E_b_ value decreased from 1120% to 192%, while in the case of the filled compositions, the elongation at break after thermooxidative aging did not exceed 214%. The aging index reached a low value (0.026–0.124) which indicates a very poor aging resistance of the vulcanizates produced. Rapid changes in strength properties after thermooxidative aging of all vulcanizates were related to the accelerated degradation of rubber macromolecules caused by the application of the substance containing an element belonging to the transition group. The iron occurs in several oxidation states, and the change in the oxidation state is a factor accelerating vulcanizate degradation. In our previous article, the effect of metal oxide on the aging of the vulcanizates was described in detail [17].

### 3.4. Flammability of Unfilled and Filled CR/BR/Nano-Fe_2_O_3_ Vulcanizates

The rubber materials made of chloroprene and butadiene rubber filled with aerosil, ultrasil, chalcedony, or sillitin are nonflammable products, as evidenced by the very high oxygen index (OI ≥ 37.5%). The oxygen index for the unfilled sample was 31.0% (Table 9). The use of both natural and synthetic silica significantly affects the formation of an insulating boundary layer during the combustion of the filled composition. Silica can trap free radicals formed as a result of polymer degradation and thermal destruction reaction. However, according to Kashiwagi, the lower flammability of polymer/silica composites is associated to a great extend with physical processes taking place in the condensed phase than with chemical reactions in the gas phase. The balance between the density and specific surface of the filler and the viscosity of the burnt material is extremely important. The silica accumulates near the surface of the burned sample as part of the boundary layer or is immersed in the molten mass of the polymer, limiting the combustion of the material [21,22].

### 3.5. Thermal Stability of Unfilled and Filled CR/Br/Nano-Fe_2_O_3_ Vulcanizates

**Figure 7:**
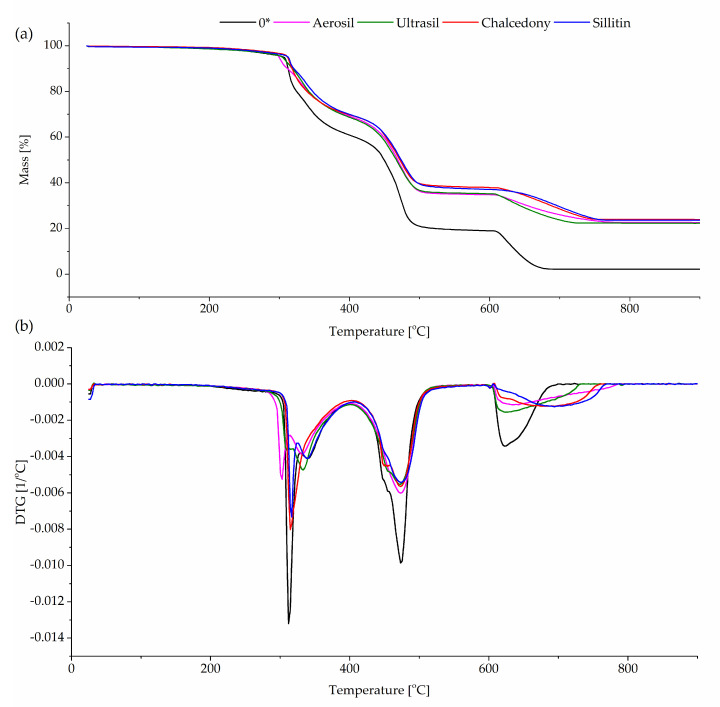
TGA (**a**) and DTG (**b**) of unfilled and filled CR/BR/nano-Fe_2_O_3_ vulcanizate.

Thermal analysis of rubber compounds was performed using thermogravimetry (Table 10, Figure 7). During heating the sample in a controlled atmosphere at constant temperature increment, changes in the mass of the test substance are observed as a result of chemical reactions (decomposition, oxidation, or reduction) and physical changes (evaporation, sublimation, desorption).

The actual thermal decomposition of the unfilled and filled CR/BR vulcanizates took place in two steps. The first thermal decomposition step of the unfilled sample began over 285°C. In this case, the weight loss was the greatest and amounted to 38.8%. The incorporation of chalcedony or sillitin did not affect the decomposition temperature in this range, it only reduces the weight loss of the sample to 30%. In the case of the elastomer composition filled with aerosil or ultrasil, the sample is pyrolyzed at a temperature of 270 °C. The first decomposition step for all samples was completed at 400 °C. The weight loss in this step was mainly due to the elimination of HCl and was seen as a large peak in the DTG curve. The less rapid course of thermal decomposition of the vulcanizates prepared took place in the second stage, which occurs in the following temperature range, 405–540 °C. The greatest weight loss (41.9%) was characteristic for the unfilled sample, while the addition of fillers reduces the value of this parameter. In the second stage of pyrolysis, the weight loss for filled vulcanizates ranged from 32.0% to 34.1%. The differences in the weight loss of samples containing fillers may be due to the thermal stability of their fillers. The presence of the large peak on the DTG curve indicates that several decomposition processes took place in this temperature range. The final stage of thermal decomposition was the carbon black formed during pyrolysis. It was found that the rate of decomposition was reduced compared to the second stage, but the wide range of temperatures of the third stage (585–790 °C) meant that the heat release in this stage of decomposition was high, which was shown on the DTA curve. Thermal decomposition of the CR/BR vulcanizates was completed at 700 °C for unfilled sample, 760 °C for the sample with chalcedony, 770 °C for the sample with sillitin, 740 °C, for the sample with ultrasil, and 790 °C for the sample with aerosil.

## 4. Conclusions

The results of our research show that the iron(III) oxide nanoparticles can be used as a crosslinking agent for the elastomeric blends containing chloroprene rubber and butadiene rubber. Additionally, the use of a nano-iron(III) oxide for crosslinking the CR/BR blends enables the conduct of interelastomeric reactions in a controlled way. The presence of additional bonds between the rubbers used contributes to obtaining vulcanizates characterized by good mechanical properties and very high resistance to fire.

The results of the conducted research show that chalcedony or sillitin can be used as alternative fillers in the elastomer technology. The dynamic tests exhibit that the occurrence of the filler–filler or filler–rubber interactions depends on the type of filler used. Ultrasil is able to create its own structure in the elastomeric matrix, and in the case of a sample containing aerosil it is possible to create filler–rubber interactions. The incorporation of sillitin or chalcedony only leads to an increase in the storage modulus. The possibility of creating filler–filler or filler–rubber interactions in the elastomeric matrix affects the functional properties of the vulcanizates produced. The CR/BR blend filled with ultrasil was characterized by the highest increase in the torque after 30 min of heating. The presence of sillitin or chalcedony contributes to the production of vulcanizates with a similar degree of cross-linking. In the case of mechanical properties, it was observed that the presence of aerosil significantly increased the tensile strength (TS_b_ = 19.80 MPa). The incorporation of the fillers enables the production of fire-resistant rubber materials, as evidenced by the high oxygen index (OI > 37.5%). Unfortunately, these vulcanizates show poor resistance to aging factors, but after the incorporation of antiaging substance, the composition designed in this way can be used for the production of many rubber materials. First of all, their increased resistance to fire allows them to be used for the production of protective clothing, fire seals, or conveyor belts. Additionally, the undoubted advantage of the presented technology is its simplicity and low manufacturing cost of incombustible materials.

## Figures and Tables

**Table 1:** The characteristics of the rubbers.

Symbol	Trade Name	Company	Content of Bound Chlorine (%)	Content of *cis* Structure	Mooney Viscosity (100 °C) (ML)	Density (g/cm^3^)
CR	Baypren^®^216	Lanxess GmbH, Dormagen, Germany	~40	5–13	38–48	1.23
BR	SYNTECA^®^44	Synthos S.A., Oswiecim, Poland	-	~97	39–49	0.91

**Table 2:** The characteristic of the fillers.

Filler	Trade Name	Company	Specific Surface Area (BET), (m^2^/g)
Sillica	Ultrasil^®^7000GR	Evonik Industries AG, Essen, Germany	175
	Aerosil^®^380	Evonik Industries AG, Essen, Germany	380
Chalcedony	Crusil M12	Crusil Co., Inowlodz, Poland	10
Neuburg siliceous earth	Sillitin Z86	Hoffmann Mineral, Neuburg (Donau), Germany	12

**Table 3:** Formulation of the elastomeric blends.

Component	Component Content (phr)			
CR	80			
BR	20			
Stearic acid	1			
Aerosil	30	-	-	-
Ultrasil	-	30	-	-
Chalcedony	-	-	30	-
Sillitin	-	-	-	30
Nano-Fe_2_O_3_	2.5			

**Table 4:** Payne effect of unfilled and filled CR/BR/nano-Fe_2_O_3_ vulcanizates.

	0*	Aerosil	Ultrasil	Chalcedony	Sillitin
**ΔG [Pa]**	210,865	332,015	898,076	242,744	397,508
**G′_max_ [Pa]**	238,314	333,081	899,439	247,517	399,321
**G″_max_ [Pa]**	31,961	68,170	138,001	38,860	57,512

ΔG′—Payne effect, G″_max_—maximum value of storage modulus, G″_max_—maximum value of loss modulus, 0*—unfilled vulcanizate.

**Table 5:** Hysteresis losses and Mullins effect of unfilled and filled CR/BR/nano-Fe_2_O_3_ vulcanizates.

	0*	Aerosil	Ultrasil	Chalcedony	Sillitin
ΔW_i_ (Nmm)	38.1	550.8	179.0	86.3	90.3
E_M_ (%)	18.5	77.1	70.0	27.5	45.3

ΔW_i_—hysteresis losses, E_M_—Mullins effect, 0*—unfilled vulcanizate.

**Table 6:** The cure characteristic of unfilled and filled CR/BR/nano-Fe_2_O_3_ composites.

Filler Type (30 phr)	t_02_ (min)	t_90_ (min)	M_min_ (dNm)	∆M_30_ (dNm)	CRI (min^−1^)	Q_v_^T^ (mL/mL)	Q_v_^H^ (mL/mL)
0*	7.30	16.70	0.68	5.93	10.64	6.20 ± 0.11	0.86 ± 0.03
Aerosil	0.06	34.31	6.17	15.76	2.92	3.98 ± 0.15	0.73 ± 0.02
Ultrasil	1.10	14.00	3.23	21.49	7.75	6.21 ± 0.09	0.83 ± 0.04
Chalcedony	3.78	11.94	0.72	9.23	12.25	6.61 ± 0.13	0.91 ± 0.06
Sillitin	4.70	11.10	0.84	9.19	15.63	6.66 ± 0.22	1.00 ± 0.03

t_02_—scorch time, t_90_—cure time, M_min_—minimal torque, ΔM_30_—increment of torque after 30 min of heating, CRI—cure rate index, Q_v_^T^, Q_v_^H^—equilibrium swelling degree in toluene or heptane, 0*—unfilled blend.

**Table 7:** Selected properties of unfilled and filled CR/BR/nano-Fe_2_O_3_ vulcanizates.

Filler Type (30 phr)	S_e100_ (MPa)	S_e200_ (MPa)	S_e300_ (MPa)	TS_b_ (MPa)	E_b_ (%)	TS_b_′ (MPa)	E_b_′ (%)	K (-)	HA (°ShA)
0*	0.70 ± 0.02	0.99 ± 0.03	1.28 ± 0.04	9.76 ± 0.40	1120 ± 26	1.50 ± 0.11	192 ± 15	0.026	30.0 ± 0.5
Aerosil	4.93 ± 0.23	7.78 ± 0.31	10.60 ± 0.40	19.80 ± 0.21	650 ± 27	11.40 ± 0,35	140 ± 10	0.124	65.1 ± 0.1
Ultrasil	1.47 ± 0.06	2.04 ± 0.04	2.67 ± 0.03	7.06 ± 0.26	688 ± 35	3.12 ± 0.09	42 ± 5	0.029	55.2 ± 0.4
Chalcedony	0.98 ± 0.07	1.56 ± 0.06	2.16 ± 0.07	8.22 ± 0.36	845 ± 30	3.05 ± 0.20	138 ± 8	0.061	36.5 ± 0.3
Sillitin	1.08 ± 0.05	1.70 ± 0.06	2.31 ± 0.05	15.30 ± 0.82	1145 ± 35	4.19 ± 0.28	214 ± 16	0.051	41.9 ± 0.7

S_e100_, _200, 300_—stress at elongation of 100%, 200%, 300%, TS_b_, TS_b_**′**—tensile strength before and after thermooxidative aging, E_b,_ E_b_**′**—elongation at break before and after thermooxidative aging, K—aging index, HA—hardness, 0*—unfilled vulcanizate.

**Table 8:** Selected properties of unfilled and filled CR/BR/nano-Fe_2_O_3_ vulcanizates.

Filler Type (30 phr)	T_s_ (N/mm)	T_τw_ (%)
0*	1.99 ± 0.06	10.00
Aerosil	12.60 ± 0.70	25.83
Ultrasil	8.16 ± 0.16	27.07
Chalcedony	3.93 ± 0.33	31.05
Sillitin	5.11 ± 0.23	28.71

T_s_—tear resistance, T_τw_—damping relative, 0*—unfilled vulcanizate.

**Table 9:** Flammability of unfilled and filled CR/BR/nano-Fe_2_O_3_ vulcanizates.

Sample	0*	Aerosil	Ultrasil	Chalcedony	Sillitin
OI (%)	31.0	>37.5	>37.5	>37.5	37.5

OI—oxygen index.

**Table 10:** Thermal stability of unfilled and filled CR/BR/nano-Fe_2_O_3_ vulcanizates.

Filler Type (30 phr)	Temperature (°C)	Weight Loss (%)
0*	285–400	38.8
	405–512	41.9
	600–700	16.9
Aerosil	270–400	30.6
	405–522	34.1
	600–790	12.3
Ultrasil	270–400	31
	405–520	33.3
	600–740	12.9
Chalcedony	290–400	29.8
	405–540	32
	590–760	14
Sillitin	290–400	29.9
	405–520	32.4
	585–770	13.6

## Data Availability

Not applicable.
